# Entropy in Image Analysis III

**DOI:** 10.3390/e23121648

**Published:** 2021-12-08

**Authors:** Amelia Carolina Sparavigna

**Affiliations:** Department of Applied Science and Technology, Polytechnic University of Turin, 10129 Turin, Italy; amelia.sparavigna@polito.it

Image analysis basically refers to any extraction of information from images, which can be as simple as QR codes required in logistics and digital certifications or related to large and complex datasets, such as the collections of images used for biometric identification or the sets of satellite surveys employed in the monitoring of Earth’s climate changes. At the same time, image analysis is necessary for providing methods for hiding information too. When images are playing a major role in data transmission, it is imperative to protect them. Therefore, increasingly sophisticated algorithms, supported by specific artificial intelligence methods as well, are indispensable in encryption and decryption of images for being up to date with secure performances in their transmission.

Regarding the image analysis involved in computer vision, we are generally used to comparing it with the human visual system and its ability of extracting high-level and relevant information. However, we currently have many applications which can be properly managed only by means of computers. Let us consider, for instance, the face recognition applied to find a profile in a huge database. This is an impossible task for humans alone, but computers are turning it into a possible one. Consequently, “image analysis” is not just what we can imagine by taking as a model our human vision system; it is all the bulk of methods that computers are using today in several multivariate applications and the body of knowledge that they will be able to manage, in future, in a totally unsupervised manner thanks to their artificial intelligence.

Together with encryption and decryption, the use of neural networks and machine learning is one of the leitmotifs contained in the articles proposed in this Special Issue, such as in the previously published Issues regarding Entropy in Image Analysis.

Let us describe the articles of the Issue shortly.

In [1], the problem of tomographic image reconstruction is addressed. In the proposed method, an extended class of power-divergence measures, which are including a large set of distances and relative entropy measures, are involved in an iterative reconstruction algorithm. The authors introduced in the method a system of nonlinear differential equations based on Lyapunov functions. Actually, the resulting iterative algorithm proposed in [1] represents a natural extension of the maximum-likelihood expectation-maximization method. 

As told before, the secure transmission of digital images in the current network and big data environment is one of the main tasks of image analysis. This is the problem considered in Ref. [2]. In their article, the authors propose a security-enhanced image communication scheme, requiring cellular neural network (CNN) and cryptanalysis. The features of CNN are applied to create pseudorandom sequences which are used in the image encryption. By means of a plain image, the cipher image is obtained in the CNN-based sequence. In [2], cryptanalysis demonstrates the safety of the performance.

In [3], modified Hilbert space-filling curves, related to rectangles and cuboids, are applied in an entropy coding of images and for video compression. By means of these Hilbert curves, an efficient run-length-based entropy coding has been developed, which is suitable for a series of high-efficiency image compression algorithms. As observed by authors, the 2-D Hilbert curves are defined on squares while subband image compression requires rectangles of arbitrary sizes. In [3], the authors provide details about the construction of the required modified 2-D Hilbert curves and 3-D cuboids. 

The word “retinex” comes from “retina” and “cortex”, since both the eyes and brain are involved in human vision. Then, in image analysis, retinex methods are those which mimic how human beings perceive their surrounding environment. In [4], we find proposed a retinex fast algorithm to enhance low-light images in order to restore the information which is hidden by low illuminance. The experimental results proposed in [4] demonstrate that the images, enhanced by the proposed retinex method, have better performance with respect to those obtained by means of other state-of-the-art methods. 

As asserted in [5], automated video surveillance systems are offering today some solutions to avoid any human intervention, which could result in inefficient tasks. In this framework, properly devised methods and models are strictly necessary. If we need a crowd surveillance, for instance, it is fundamental to analyze the human crowd behavior (HCB) by means of systems possessing robust feature extraction methods and reliable decision-making classifiers. In [5], the authors describe an approach based on a particles force model for multi-person tracking, the performance of which has been tested on publicly available benchmark datasets. 

In [6], we find the methodology referred to as full waveform inversion (FWI) applied to subsurface investigations. This methodology is commonly used in the petroleum industry, mainly to characterize oil reservoirs. The authors of [6] propose the addition of a relative entropy in the formalism of FWI. In the article, the authors show some features of this entropy and propose three different ways to add information through it in the inverse problem. This prior information, conveyed by the addition of entropy relative to FWI, can provide a result with better resolution. 

Diagnostic radiography designates a technical mode of acquiring medical images by means of X-rays. In the used devices, an electron beam is converted into X-rays by means of a target material. As a result of the mechanisms of emission, the field intensity towards the cathode is larger than the intensity towards the anode. This is the so-called anode heel effect, addressed in [7], intended to cause a non-uniform image quality. The purpose of the study proposed in [7] is that of evaluating the non-uniformity in digital radiographs. The author is also giving a novel method, based on circular step-wedge phantom and normalized mutual information, which outperforms the conventional visible ratio metrics. 

In Ref. [8], we can find again the problem of image security. According to the proposed discussion, hyperchaotic image encryption is the method which is today generally used to secure images. In this framework, article [8] is proposing a novel encryption scheme based on multiple bit permutation and diffusion (MBPD). The method is described in detail, starting from a four-dimensional hyperchaotic system with Lyapunov exponents and ending with permutation and diffusion. After experiments, it is concluded that MBPD can effectively resist different types of attacks with better performance than popular encryption methods. 

A computerized tomography (CT) scan is a medical imaging technique which combines a series of X-ray images taken from different angles around the body, processing them to create cross-sectional images (slices) of it. In [9] we can find CT scan used to evidence the spleen injuries, and an automated method based on machine learning for processing it. In fact, computer-assisted diagnosis systems exist for other conditions, but for spleen injuries the current methodology is based on detecting them by manual inspection. The results proposed by [9] suggest that a quantitative computerized analysis of spleen injuries can help in providing a faster triage (with a consequent improvement of patient outcomes).

In [10], we can find another method addressing the protection of digital information, in particular the digital visual information. The method is based on a six-dimensional hyper-chaotic encryption scheme and a three-dimensional transformed Zigzag diffusion with RNA operation. With respect to the previous literature, the specificity of the method proposed in [10] is in its focusing on the encryption of color images. The encryption starts with three pseudo-random matrices generated from a 6D hyper-chaotic system. It continues with a permutation and a transformation by means of Zigzag diffusion. The final step is RNA conversion. Experiments show that the proposed encryption has high resistance against generally used attacks. 

The fractional calculus, made by means of operators of non-integer order, is mainly used in the area of nonlinear and chaotic systems. In [11], we can find it in a fractional-order hyperchaotic system applied to secure communication, in particular for the encryption of color images. Experiments reported in [11] are supporting the method as cryptographically secure in general. However, the method can be broken in some cases. Therefore, the final suggestion given by authors is that algorithm designers have to pay some specific attention in securing this kind of cryptosystems. 

Article [12] explains that, to encrypt/decrypt images, most researchers are using chaotic systems, whereas others prefer non-chaotic methods. In [12], a new encryption algorithm is proposed, which combines a non-chaotic Newton-Raphson’s method with a hyperchaotic two-dimensional map of a general Bischi-Naimzadah duopoly system. The multiple security experiments made for measuring the efficiency of the method (among which we find entropy analysis) show that the proposed algorithm possesses a good security efficiency.

In concluding the review of the Issue, a great interest in image encryption and decryption has been demonstrated. However, as shown by the different topics and problems addressed in the other published articles, the research field of image analysis is quite larger and variegated and not solely limited to problems of cryptanalysis. Therefore, the guest editor hopes that the readers can receive, from these published articles, fruitful hints and inspirations for future research and publications, of which the Topical Collection “Entropy in Image Analysis” (https://www.mdpi.com/journal/entropy/special_issues/entropy_image_TC, accessed on 5 December 2021) could represent the proper publication place.
